# Safety of sinopharm COVID-19 vaccine in children and adolescents aged 5 to 18 years: a cohort event monitoring study

**DOI:** 10.1186/s12889-025-25308-1

**Published:** 2025-11-28

**Authors:** Mohammad Hassan Emamian, Sajad Sahab-Negah, Sairan Nili, Parvin Mangolian shahrbabaki, Alireza Ansari-moghaddam, Mohammad Fereidouni, Mostafa Enayatrad, Sepideh Mahdavi, Roqayeh Aliyari, Mansooreh Fateh, Hamidreza Khajeha, Zahra Emamian, Elahe Behmanesh, Hamid Sharifi

**Affiliations:** 1 Ophthalmic Epidemiology Research Center, Shahroud University of Medical Sciences, Shahroud, Iran; 2 Multiple Sclerosis Research Center, Neuroscience Institute, Tehran University of Medical Sciences, Tehran, Iran; 3 Social Determinants of Health Research Center, Research Institute for Health Development, Kurdistan University of Medical Health, Sanandaj, Iran; 4 Nursing Research Center, Razi Faculty of Nursing and Midwifery, Department of Critical Care, Kerman University of Medical Sciences, Kerman, Iran; 5 Health Promotion Research Center, Zahedan University of Medical Sciences, Zahedan, Iran; 6 Cellular and Molecular Research Center, Birjand University of Medical Sciences, Birjand, Iran; 7 Clinical Research Development Unit, Bahar Hospital, Shahroud University of Medical Science, Shahroud, Iran; 8 Department of Epidemiology, School of Public Health, Shahroud University of Medical Sciences, Shahroud, Iran; 9 Center for Health Related Social and Behavioral Sciences Research, Shahroud University of Medical Sciences, Shahroud, Iran; 10 Health Technology Incubator Center, Shahroud University of Medical Sciences, Shahroud , Iran; 11 HIV/STI Surveillance Research Center, and WHO Collaborating Center for HIV Surveillance, Institute for Futures Studies in Health, Kerman University of Medical Sciences, Kerman, Iran

**Keywords:** BBIBP CorV, Adolescent, Child, Longitudinal studies, COVID-19

## Abstract

**Background:**

Although the safety of the BBIBP-CorV (Sinopharm) vaccine has been confirmed in both adults and children, safety data remain limited for individuals under 18 years of age. This longitudinal study in Iran aimed to investigate reactogenicity, breakthrough infections, and serious adverse events following Sinopharm vaccination in this population.

**Methods:**

In this cohort event monitoring study, children and adolescents aged 5 to 18 Years in the eight cities around the country were invited to participate after receiving the first dose of the vaccine. The assessment of local and systemic reactogenicities was conducted through daily telephone calls for up to seven days following the first and second doses of the vaccine. Severe adverse events, adverse events of special interest (AESI), and COVID-19 infections were monitored with weekly follow-ups. Follow-up lasted for 13 weeks for those who received one dose of the vaccine and 17 weeks for those who received two doses. Adverse events were reported as percentages along with 95% confidence intervals (CI). The total person-time at risk was used to calculate the incidence rate of COVID-19 infections, and factors associated with COVID-19 were analyzed using Cox survival analysis.

**Results:**

Among the 18,528 participants (50.5% female), the mean age was 11.9 ± 3.4 years (age range: 4.6–18.6). Only 57.4% of participants received the second dose of the vaccine. The incidence rate of COVID-19 infection was 28.9 (95% CI: 26.4–31.6) per 100,000 person-days. Two cases were classified as AESI: one case of generalized convulsion and one case of diabetic ketoacidosis. Reactogenicity was investigated in a subset of 945 participants, 184 of whom received the second vaccine dose. The most frequent local and systemic reactogenicity events on the first day after vaccination were as follows: pain at the injection site—26.7% (95% CI: 23.5–29.8) after the first dose and 53.3% (95% CI: 46.0–60.5) after the second dose; headache—6.0% (95% CI: 4.4–7.7) after the first dose and 8.7% (95% CI: 4.6–12.8) after the second dose; fatigue—3.7% (95% CI: 2.3–5.0) after the first dose and 7.1% (95% CI: 3.4–10.8) after the second dose; and malaise—2.9% (95% CI: 1.7–4.1) after the first dose and 7.6% (95% CI: 3.8–11.5) after the second dose. Most reactogenicity events subsided gradually and did not interfere with the participants’ daily activities.

**Conclusions:**

Sinopharm is a safe and well-tolerated vaccine option for children and adolescents and can be recommended for this population.

## Background

From the beginning of the COVID-19 pandemic to February 2, 2025, there have been 777.4 million confirmed cases globally, causing more than 7 million deaths [1]. Although significant progress has been made in COVID-19 management, it remains a concern for pediatric populations due to three key factors: (I) children remain susceptible to severe illness [2]; (II) they can transmit the virus to others, including vulnerable populations [3]; and (III) lower vaccination rates coupled with vaccine hesitancy [4, 5] may contribute to increased transmission, potential emergence of new variants, and added strain on healthcare systems. Effective efforts were made toward vaccine development by various countries. As of March 23, 2023, out of 183 vaccines in the clinical phase, 11 had passed various stages of safety and efficacy evaluation and were in the fourth phase of clinical trials [6].

The majority of COVID-19 vaccines are approved only for use in adults, with almost all clinical trials assessing vaccine safety and effectiveness conducted on individuals over 18 years old. Gradually, as sufficient evidence of vaccine safety emerged, their use was extended to individuals under 18 years of age. The SARS-CoV-2 Vaccine (Vero Cell), Inactivated (lnCoV) (Sinopharm, BBIBP CorV) was one of the first inactivated virus-based vaccines initially licensed for adults. Subsequently, Chinese authorities approved this vaccine for individuals aged 3 to 17 years [7]. However, there is limited information available regarding the safety and adverse events of the vaccine in children and adolescents. Although a few studies [8–14] have evaluated the safety of the Sinopharm vaccine in this age group, they suffer from methodological issues, including lack of longitudinal design, small sample sizes, and failure to systematically investigate all complications.

A cohort event monitoring (CEM) study for safety signal detection after vaccination with COVID-19 vaccines in Iran [15] was conducted using a template provided by the World Health Organization (WHO) [16]. The study offered valuable evidence regarding the safety of four COVID-19 vaccines: Sputnik V, Sinopharm, CoVIran Barkat, and AZD1222. Given the limited evidence regarding the safety of COVID-19 vaccines in children and adolescents, this study focused on in children and adolescents aged 5 to 18 Years who received the Sinopharm. The objective was to investigate reactogenicities, Serious Adverse Events (SAEs), the COVID-19 breakthrough rate, and Adverse Events of Special Interest (AESIs) in children and adolescents aged 5 to 18 Years after receiving the Sinopharm vaccine.

## Methods

The study protocol has already been published [15]. Briefly, the study started in October 2021 and ended in July 2022, enrolling 18,528 participants aged 5 to 18 Years. Participants were recruited among people who were vaccinated using the Sinopharm vaccine in the cities of Shahroud, Zahedan, Sanandaj, Birjand, Kerman, Mashhad, Tehran, and Kermanshah. The selection of the study sites was based solely on three criteria: the presence of tertiary hospitals capable of diagnosing AESIs, the commitment of local authorities, and the availability of eligible co-investigators. At each study site, at least one vaccination center was chosen, and all individuals who had been vaccinated were invited to participate in the study. Study participation was strictly voluntary and required the signing of written informed consent by participants and parents or legal guardians. Registration of participants continued until the desired sample size was reached. All vaccinated individuals with Sinopharm who were aged between 5 and 18 years at the study sites were eligible for inclusion in the study. Eligibility verification for vaccination was the responsibility of healthcare workers at the vaccination sites. Aside from a history of severe allergy to any vaccine component, there were no other contraindications for COVID-19 vaccination. Participants who did not sign the written consent form themselves or whose parents did not sign were excluded from the study. Contact details and demographic information, as well as a history of underlying diseases, were collected during enrollment. The vaccine dose was the same across all age groups (5 to 18 years) as that for adults: 4 µg (0.5 mL). This dosage is consistent with the interim recommendations from the World Health Organization [17] and also is supported by the clinical trial evidences [18]. The interval between the two vaccine doses was four weeks. Data collection forms and questionnaires have previously been published elsewhere [11, 12].

### Measurement of reactogenicities

According to the WHO guideline [12], the plan was to include the first 1,000 individuals enrolled in the reactogenicity (The short-term reactions that result from the body’s initial inflammatory response.) subset. These participants were followed daily for seven days after receiving the first and second doses of the vaccine. The reactogenicity of vaccines was investigated by daily phone contact with participants or self-reporting through the web application. Participants were contacted by phone up to twice daily. If they were not reachable, their next of kin was contacted. If that attempt also failed, the call was recorded as missed. The local reactogenicities included pain at the injection site, redness, swelling, induration, warmth, and itching. The systemic reactogenicities included fever, nausea, malaise, chills, headache, joint pain, myalgia, and fatigue. The proportion of individuals with local or systemic reactogenicities, along with 95% confidence intervals, was reported by vaccine doses seven days after vaccination. Multiple generalized estimating equation (GEE) models with an unstructured correlation matrix were used to compare reactogenicities between the two vaccine doses over the seven days following vaccination. The GEE models achieved convergence using the default criteria in our statistical software (STATA), which is a relative gradient convergence criterion of 1e-8. All models converged successfully without warnings, indicating stable parameter estimates. To ensure the robustness of our findings, we conducted sensitivity analyses by fitting the models with alternative correlation structures (exchangeable, independent). The estimated coefficients and their significance levels were consistent across these different structures. The severity of reactogenicities was reported according to the interference of reactogenicities with the participants’ daily activities. Participants in the reactogenicity subset were older because the vaccination program for children under 18 began with older age groups and gradually expanded to include younger children, eventually reaching those as young as 5 years old.

### COVID-19 breakthrough infection

Each enrolled participant actively followed up until three months after his or her last COVID-19 vaccine dose. The questionnaires were completed through telephone calls by trained interviewers or by using a self-reporting web-based application at weekly intervals. The diagnosis of COVID-19 infection was based on self-reported reverse transcription polymerase chain reaction (PCR) or antigen rapid tests. Medical hospital records were also checked for unreliable or unavailable responses. The incidence rate of COVID-19 disease was calculated by dividing the total number of cases by the total person-days followed up. The incidence rates were reported with 95% confidence intervals (CI). A multiple Cox proportional hazards regression model was used to assess the factors associated with COVID-19 disease after vaccination, adjusting for age, sex, prior COVID-19 infection, and comorbidities. Calendar time was used as the timescale to ensure the proportional hazards assumption in the Cox regression model. The entry date for calculating the time to events was set to 14 days after vaccination to exclude the first two weeks following vaccination from the analysis. The significance level was considered ≤ 0.05. Participants were considered censored if they experienced a COVID-19 infection, reached the end of the follow-up period, or were lost to follow-up. The follow-up index (FUI) was calculated by dividing the actual investigated follow-up period by the potential follow-up duration [13].

### Adverse events of special interest and serious adverse events

The AESIs included 22 different outcomes that may occur following COVID-19 vaccination [11]. Any adverse events following vaccination that needed overnight hospitalization of vaccinated individuals were considered “serious”. All hospitalized participants underwent comprehensive evaluations by classification committees at each study site for AESIs. These committees included specialists in infectious disease, immunology, epidemiology, internal medicine, cardiology, clinical pharmacology, and other relevant fields. Their responsibility was to review medical records and assess whether the adverse events were associated with the COVID-19 vaccines.

## Results

**Fig. 1:**
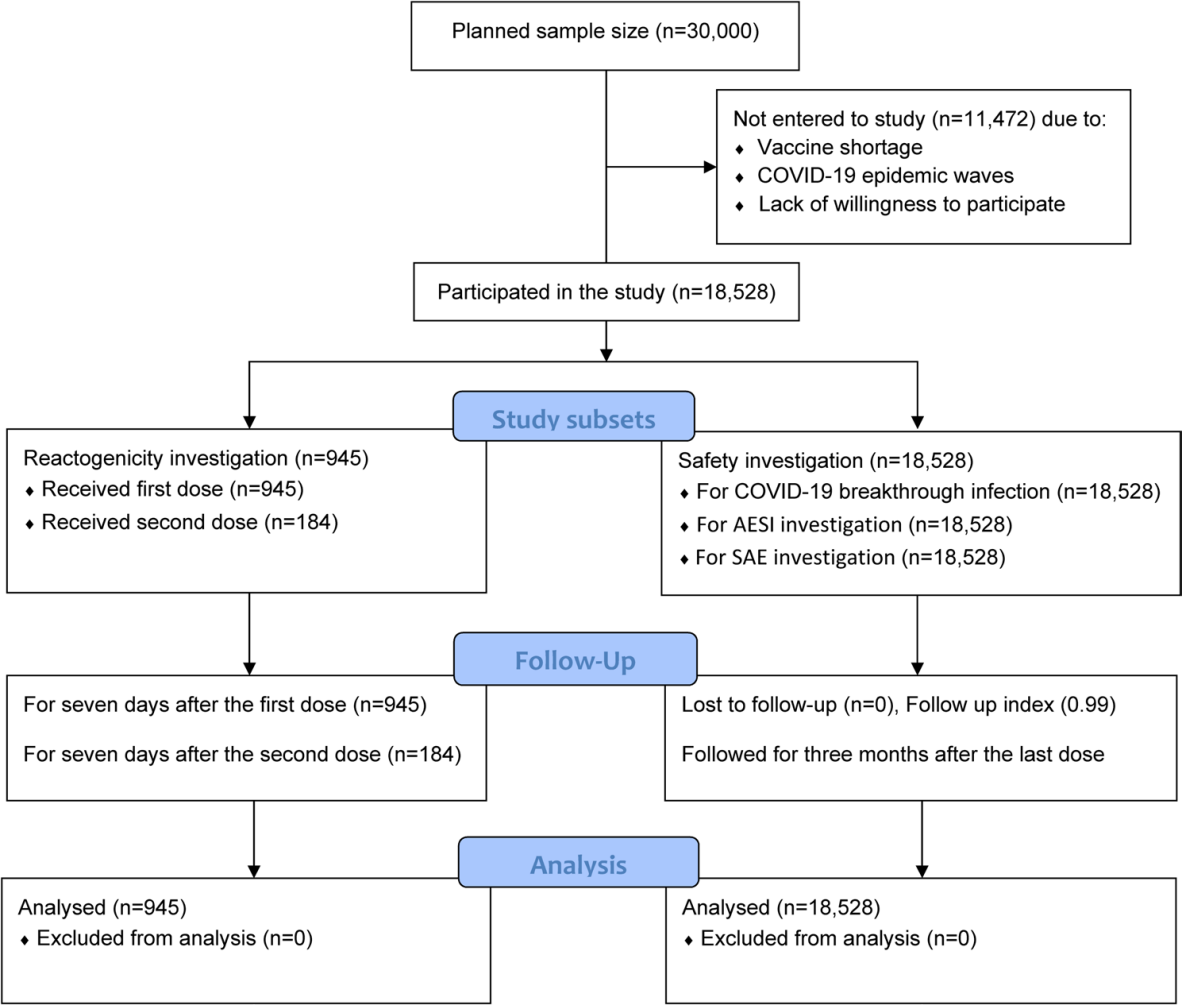
The flow diagram of participants in two study groups

**Fig. 2:**
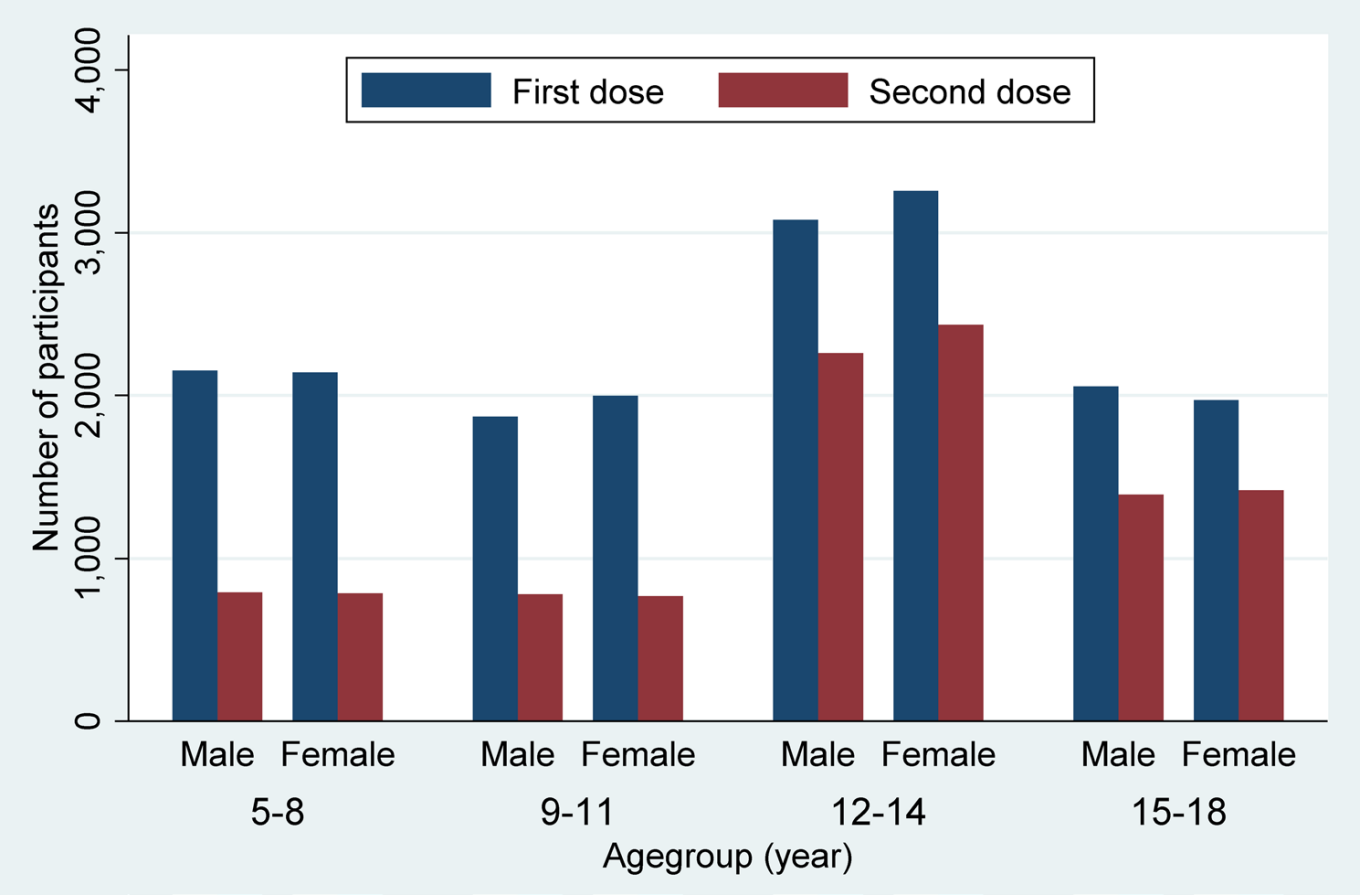
The frequency of enrolled participants by sex, age groups, and vaccine doses

Among the 18,528 participants, the mean age of participants was 11.9 (SD = 3.4 years). The age range was between 4.6 and 18.6 years, and 50.5% of participants were female. Among the included participants after the first dose, 10,642 (57.4%) of participants received the second dose of the vaccine (Fig. 1). The mean (SD) follow-up duration was 106.2 (15.6) days, and the follow-up index was 0.9919 (0.068), indicating a very low loss to follow-up rate. The follow-up index values were 0.9964, 0.9932, 0.9910, and 0.9893 for the age groups 5–8, 9–11, 12–14, and 15–18 years, respectively. Additionally, the index remained high after the first dose (0.9907) and the second dose (0.9929) of the vaccine. Figure 2 shows the distribution of participants by age and sex groups. Table 1 compares the baseline characteristics of participants according to the number of vaccine doses received. Participants who received both doses were older, had a higher prevalence of prior COVID-19 infection, and had a lower percentage of renal disease compared to those who received only one dose.

**Table 1:** Comparison of baseline characteristics between participants who received both doses of the vaccine and those who received only the first dose

Independent variables		Receiving both doses of vaccine (*n* = 10642)	Receiving only first doses of vaccine (*n* = 7886)	*P* value
Age (year), mean (SD)		12.8 (3.2)	10.8 (3.5)	< 0.001†
Male sex, (n, (%))		5228 (49.1)	3930 (49.8)	0.340‡
Prior COVID-19 infection (n, (%))		1226 (11.5)	628 (8.0)	< 0.001‡
History of comorbidities (n, (%))	Reparatory diseases	68 (0.64)	54 (0.68)	0.703‡
	Cardiac diseases	28 (0.26)	23 (0.29)	0.714‡
	Allergy	146 (1.37)	112 (1.42)	0.781‡
	Renal diseases	11 (0.10)	22 (0.28)	0.005‡
	Diabetes	17 (0.16)	16 (0.20)	0.491‡
	Neurologic diseases	33 (0.31)	15 (0.19)	0.112‡

*SD* Standard deviation, † t-test, ‡ Chi squared test

**Fig. 3:**
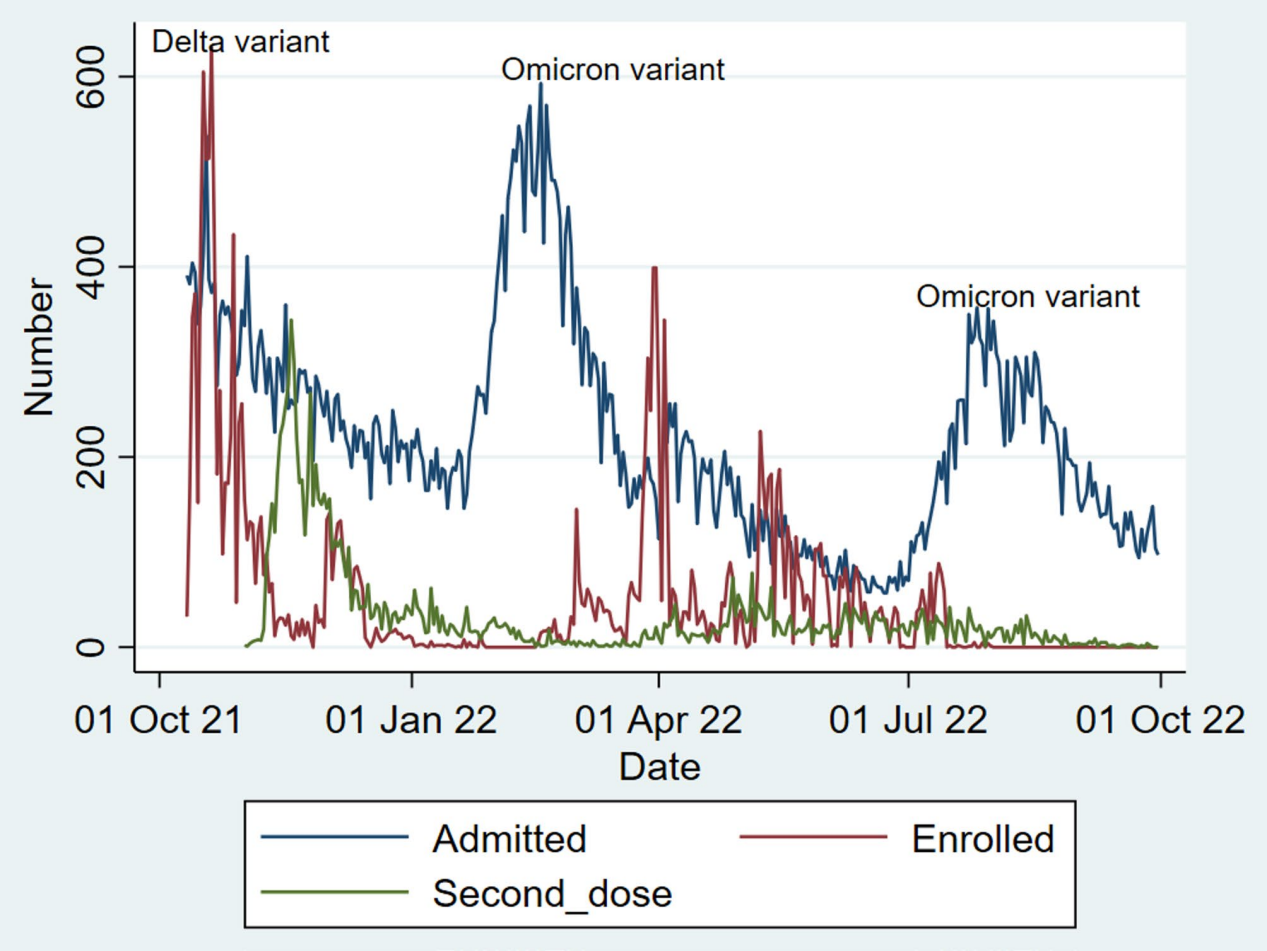
The daily number of enrolled participants and administrated second dose of vaccine in comparison with an epidemic curve, defined by daily admitted cases of COVID-19 in studied cities

According to national reports, the dominant variants of SARS-CoV-2 were different during the study period. Most participants were enrolled in the study after the subsidence of the epidemic due to the Delta variant and then from April to July 2022, when the Omicron epidemic curve subsided (Fig. 3). This issue is important when comparing the incidence rate of COVID-19 in different studies. In total, the weekly follow-up was completed for 16,768 (90.5%) participants.

### Reactogenicity

Among 945 individuals who were investigate for reactogenicity, 761 individuals (80.5%) received only the first dose and 184 individuals (19.5%) received both doses of vaccine. The primary reasons for not reaching 1,000 participants in the reactogenicity subset were: (I) a coding error that misclassified participants over 18 years as being under 18, and (II) participant unwillingness to receive the second vaccine dose. The mean age of participants for the first dose was 14.5 (SD = 1.7; range: 10–18; 50.6% female). For the second dose, the mean (SD) of participants was 14.2 (1.8), with an age range of 11 to 18 years (50.9% female).

**Fig. 4:**
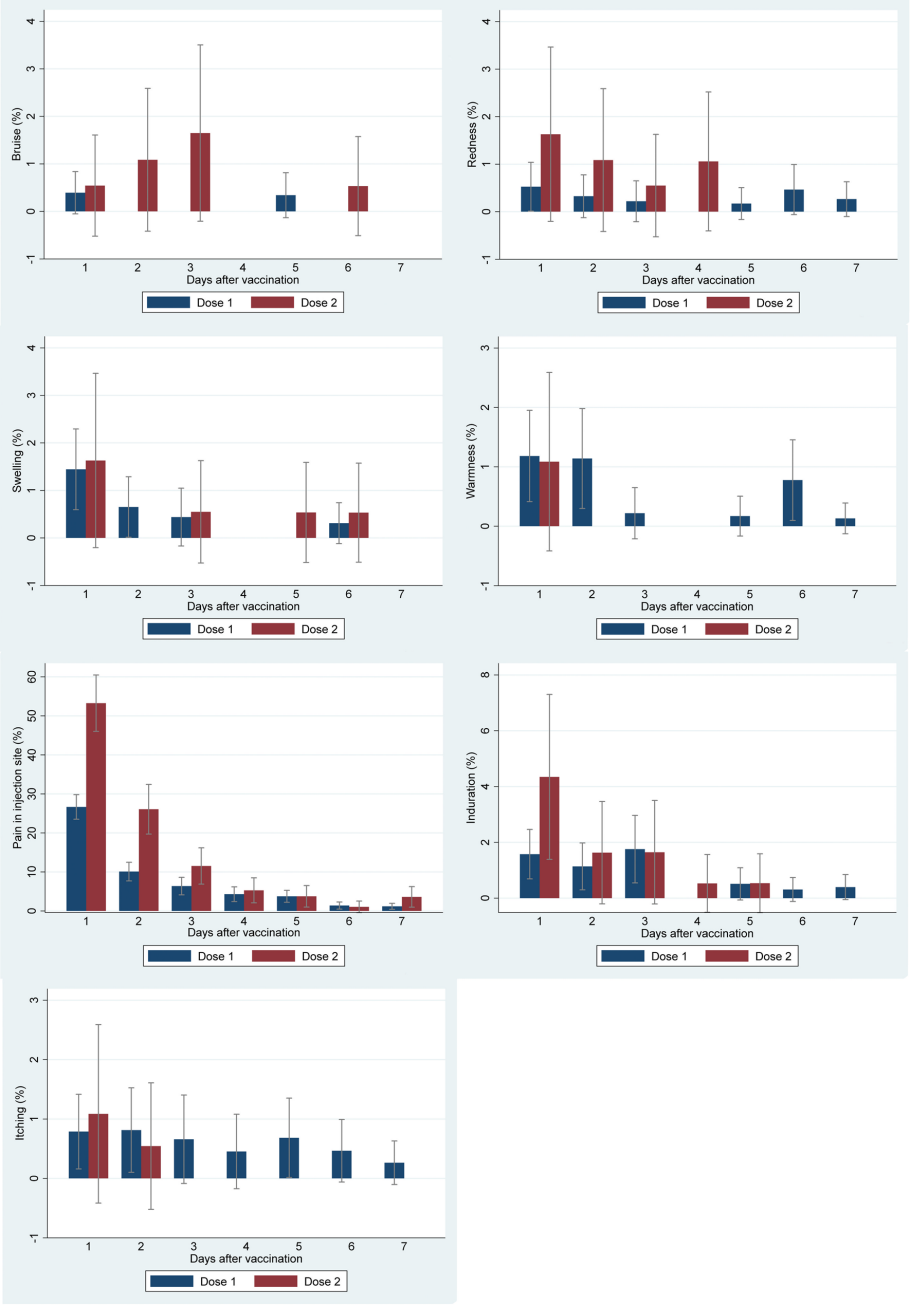
Comparison of local reactogenicities following first and second doses of Sinopharm vaccine in children

**Fig. 5:**
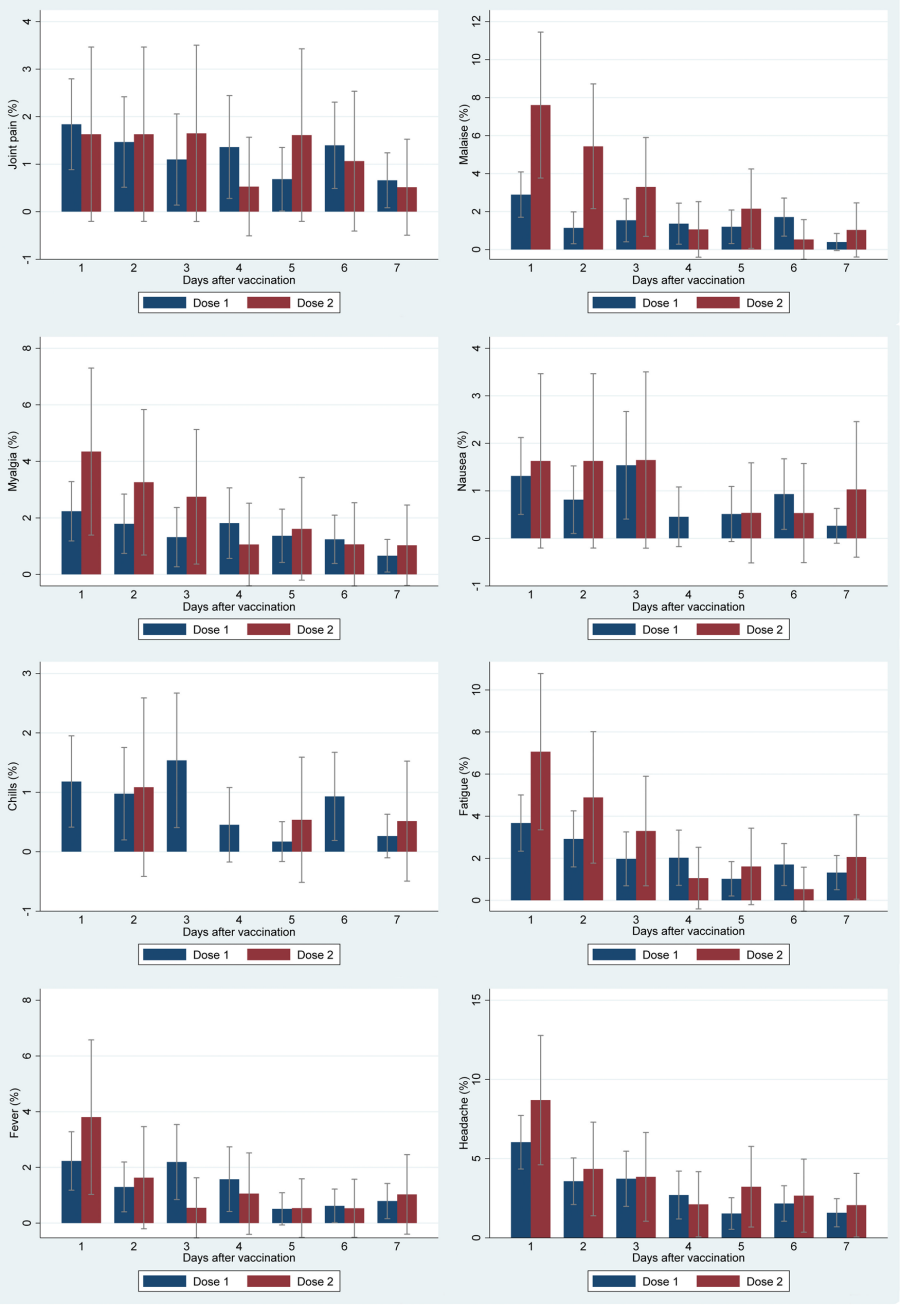
Comparison of systemic reactogenicities following first and second doses of Sinopharm vaccine in children

**Fig. 6:**
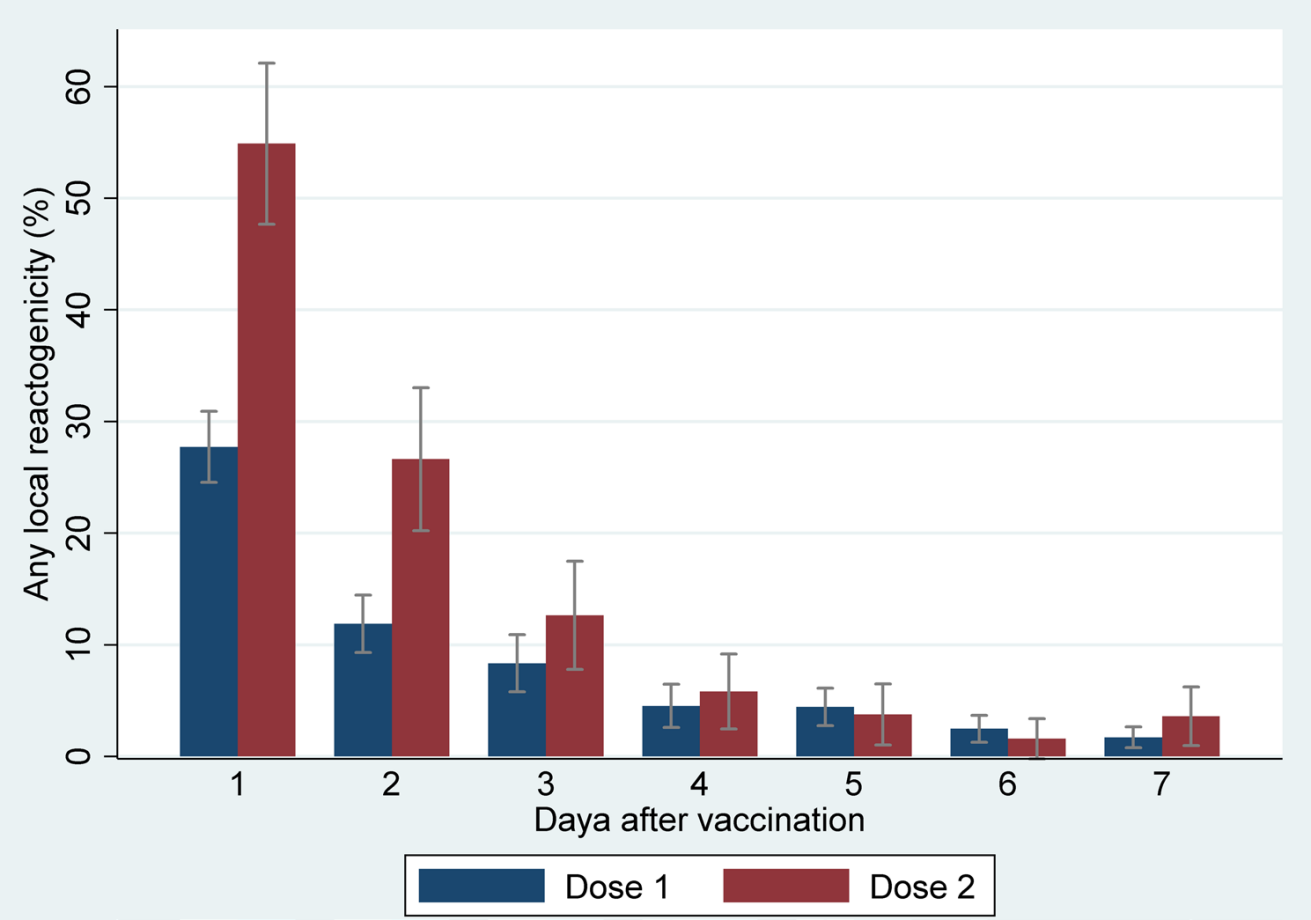
Incidence of at least one local reactogenicity following first and second doses of Sinopharm vaccine in children

**Fig. 7:**
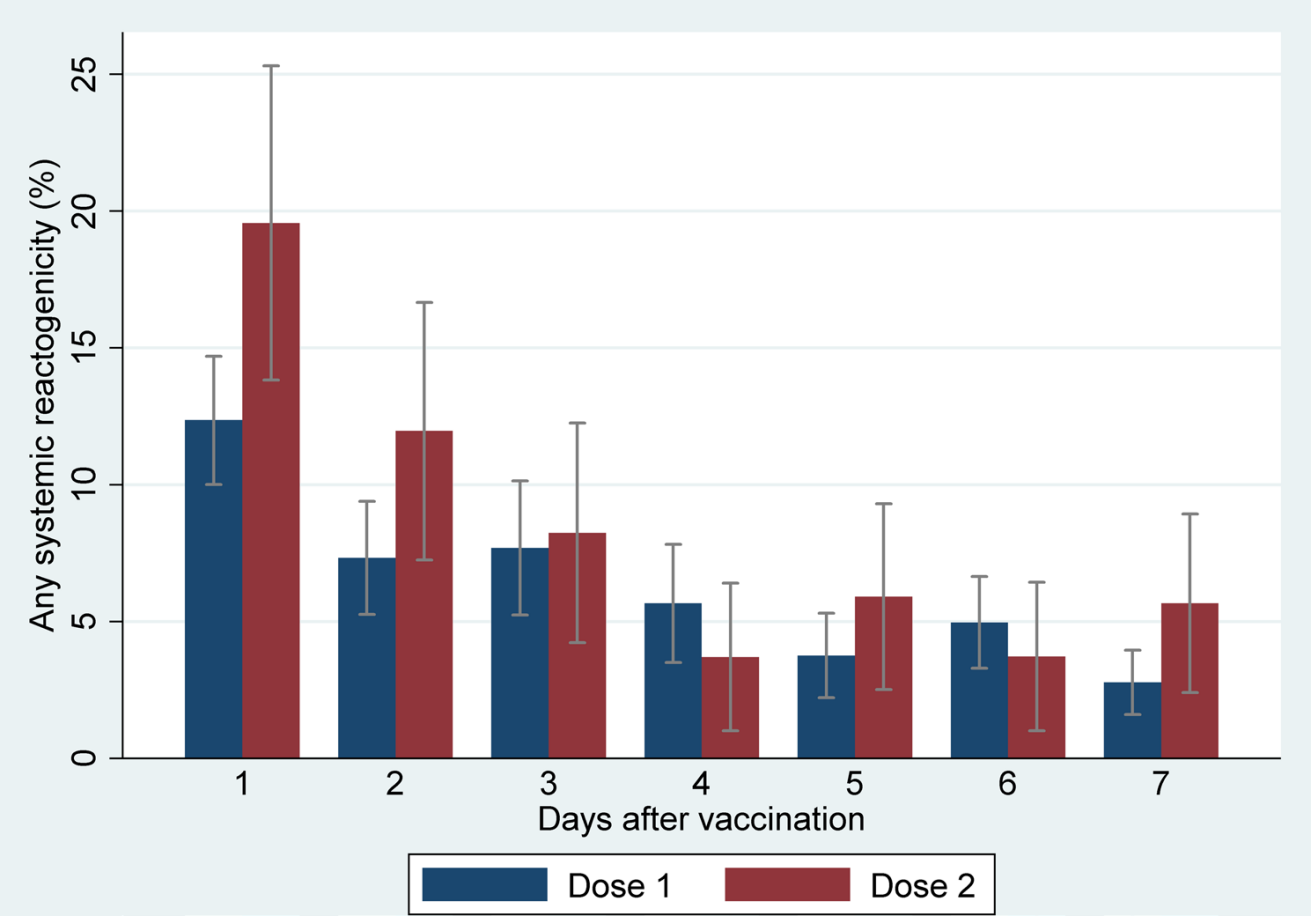
Incidence of at least one systemic reactogenicity following first and second doses of Sinopharm vaccine in children

**Fig. 8:**
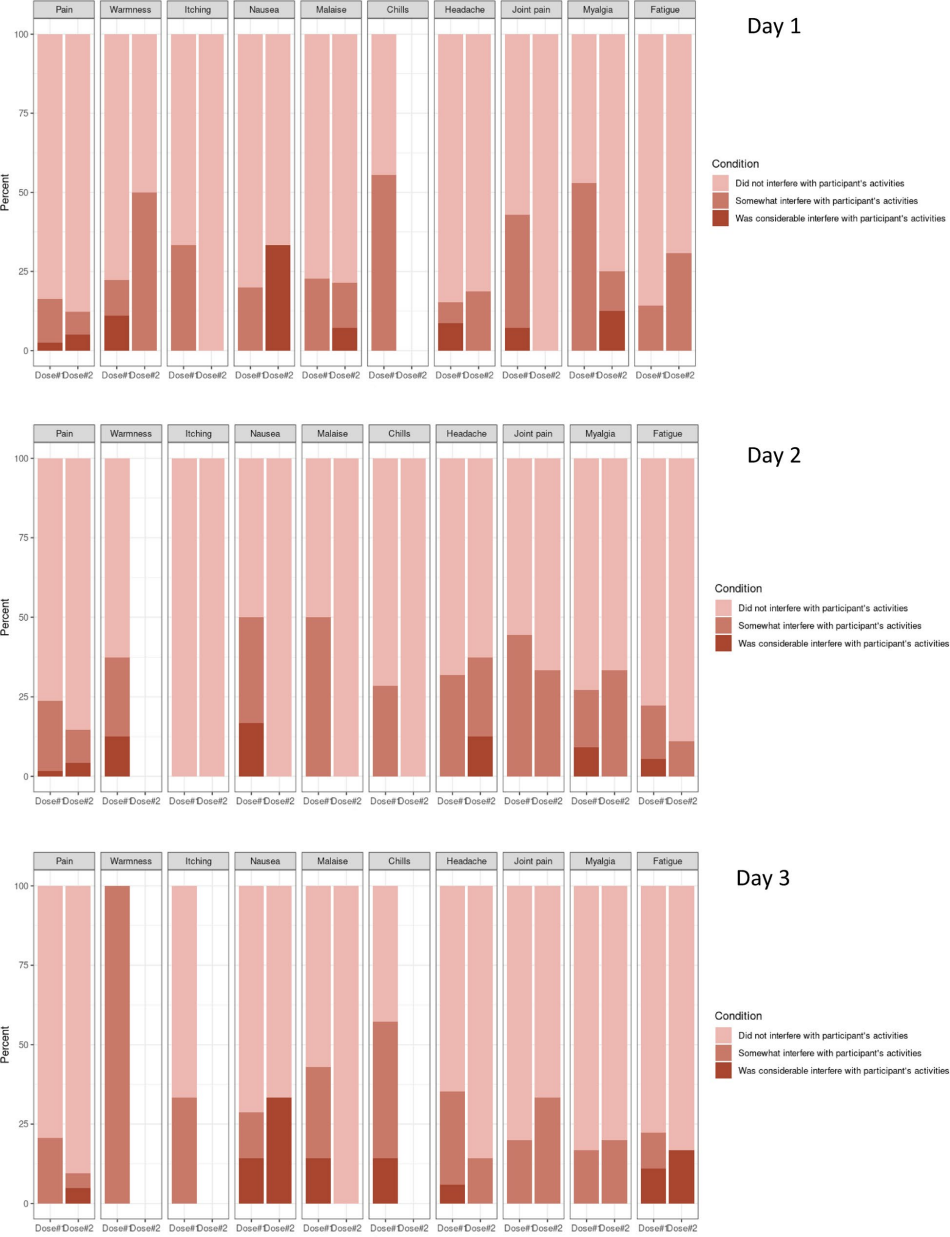
The severity of local and systemic reactogenicities among participants who reported these effects within the first three days after vaccination, categorized by vaccine dose

During the first day after vaccination, pain at the injection site (26.7% (95% CI: 23.5–29.8) after the first dose and 53.3% (95% CI: 46.0–60.5) after the second dose), headache (6.0% (95% CI: 4.4–7.7) after the first dose and 8.7% (95% CI: 4.6–12.8) after the second dose), fatigue (3.7% (95% CI: 2.3–5.0) after the first dose and 7.1% (95% CI: 3.4–10.8) after the second dose), and malaise (2.9% (95% CI: 1.7–4.1) after the first dose and 7.6% (95% CI: 3.8–11.5) after the second dose) were the most frequent local and systemic reactogenicity events observed among participants (Figs. 4 and 5). The incidence rate of most reactogenicity events increased after the second dose of the vaccine compared with the first dose; nevertheless, the differences between the reactogenicity events in the first and second doses were not statistically significant, except for the pain at the injection site in the first three days after vaccination (Figs. 4 and 5) (Tables 2 and 3), and induration and malaise on the first day after vaccination (Tables 2 and 3). In total, 27.7% (95% CI: 24.5–30.9) of the participants had at least one local reactogenicity event on the first day after vaccination, and 54.9% (95% CI: 47.7–62.1) of them had this outcome on the first day of the second dose vaccination (Table 4; Fig. 6). More detailed information on the incidence of at least one local reactogenicity, stratified by age and sex, is presented in Table 4. For systemic reactogenicities, these estimates were 13.8% (95% CI: 11.6–16.0) and 19.6% (95% CI: 13.8–25.3) for the first and the second doses, respectively (Table 5; Fig. 7). Local and systemic reactogenicities were more frequently reported among girls within the first day following the first vaccine dose (Tables 4 and 5). The incidence of local reactogenicities exhibited a decreasing trend from the first to the seventh day after vaccination (Fig. 6). A similar, though non-linear, trend was observed for systemic reactogenicities (Fig. 7). Most cases with reactogenicity reported that this event was mild and did not interfere with their daily activities (Fig. 8).

**Table 2:** The associated factors with local reactogenicity events in the 1–7 days after vaccination in generalized estimating equations (GEE) models

Adverse effects		Day 1		Day 2		Day 3		Day 4		Day 5		Day 6		Day 7	
		IRR	*P* value	IRR	*P* value	IRR	*P* value	IRR	*P* value	IRR	*P* value	IRR	*P* value	IRR	*P* value
Pain	Dose 2/Dose 1	**1.6** **(1.3–2.0.3.0)**	**< 0.001**	**2.1** **(1.5–2.9)**	**< 0.001**	**1.8 (1.0–3.2.0.2)**	**0.036**	1.3 (0.6–2.9)	0.482	1.1 (0.5–2.5)	0.849	0.7 (0.2–2.1)	0.494	**3.0 (1.1–8.1)**	**0.031**
	Age (year)	**0.9** **(0.9–1.0.9.0)**	**0.040**	0.9 (0.9–1.1)	0.484	1.1 (0.9–1.3)	0.424	1.0 (0.8–1.2)	0.773	1.2 (0.9–1.4)	0.200	1.0 (0.7–1.4)	0.916	0.9 (0.7–1.2)	0.367
	Female sex	**1.6** **(1.2–2.0.2.0)**	**0.001**	1.5 (1.0–2.3.0.3)	0.057	**2.1 (1.2–4.0.2.0)**	**0.016**	2.1 (1.0–4.5.0.5)	0.057	1.0 (0.5–2.1)	0.952	2.5 (0.6–10.9)	0.217	1.7 (0.6–4.7)	0.285
	Prior COVID-19	**1.4** **(1.1–1.7)**	**0.007**	**1.6** **(1.1–2.2)**	**0.006**	1.5 (0.9–2.4)	0.099	1.5 (0.8–2.6)	0.229	1.1 (0.5–2.5)	0.849	0.6 (0.1–3.9)	0.609	1.5 (0.7–3.3)	0.326
Redness	Dose 2/Dose 1	2.4 (0.5–10.8)	0.268	2.9 (0.4–20.8)	0.302	NC		NC		NC		NC		NC	
	Age (year)	**0.5** **(0.3–0.9)**	**0.031**	0.8 (0.4–1.3))	0.217	NC		NC		NC		NC		NC	
	Female sex	1.8 (0.4–7.8)	0.465	4.2 (0.4–40.7)	0.220	NC		NC		NC		NC		NC	
	Prior COVID-19	2.2 (0.8–6.2)	0.137	**7.4 (2.3–23.5)**	**0.001**	NC		NC		NC		NC		NC	
Swelling	Dose 2/Dose 1	1.3 (0.6–4.7)	0.701	NC		1.1 (0.1–12.5)	0.931	NC		NC		NC		NC	
	Age (year)	1.2 (0.9–1.7)	0.185	NC		0.8 (0.4–1.5)	0.427	NC		NC		NC		NC	
	Female sex	2.3 (0.7–7.4)	0.165	NC		2.1 (0.2–23.0)	0.557	NC		NC		NC		NC	
	Prior COVID-19	0.7 (0.2–2.7)	0.615	NC		1.8 (0.3–11.0)	0.553	NC		NC		NC		NC	
Induration	Dose 2/Dose 1	**2.6** **(1.1–6.5)**	**0.035**	1.5 (0.4–5.5)	0.591	1.0 (0.3–3.7)	0.991	NC		NC		NC		NC	
	Age (year)	0.9 (0.7–1.1)	0.546	1.2 (0.9–1.5)	0.230	1.0 (0.7–1.5)	0.829	NC		NC		NC		NC	
	Female sex	2.0 (0.8–5.0.8.0)	0.148	4.0 (0.9–18.7)	0.077	2.4 (0.6–9.1)	0.198	NC		NC		NC		NC	
	Prior COVID-19	1.3 (0.6–2.9)	0.546	1.2 (0.4–3.6)	0.779	0.9 (0.3–3.4)	0.914	NC		NC		NC		NC	
Bruise	Dose 2/Dose 1	1.2 (0.1–12.0)	0.860	NC		NC		NC		NC		NC		NC	
	Age (year)	0.8 (0.4–1.4)	0.370	NC		NC		NC		NC		NC		NC	
	Female sex	1.1 (0.2–8.1)	0.905	NC		NC		NC		NC		NC		NC	
	Prior COVID-19	1.4 (0.2–8.2)	0.721	NC		NC		NC		NC		NC		NC	
Warmness	Dose 2/Dose 1	1.0 (0.2–4.6)	0.973	NC		NC		NC		NC		NC		NC	
	Age (year)	1.1 (0.8–1.5)	0.685	NC		NC		NC		NC		NC		NC	
	Female sex	2.5 (0.7–9.6)	0.179	NC		NC		NC		NC		NC		NC	
	Prior COVID-19	1.3 (0.5–3.9)	0.601	NC		NC		NC		NC		NC		NC	

*NC* Not converged

**Table 3:** The associated factors with systemic reactogenicity events in the 1–7 days after vaccination in generalized estimating equations (GEE) models

Adverse effects			Day 1		Day 2		Day 3		Day 4		Day 5		Day 6		Day 7	
			IRR	*P* value	IRR	*P* value	IRR	*P* value	IRR	*P* value	IRR	*P* value	IRR	*P* value	IRR	*P* value
Fever		Dose 2/Dose 1	1.8 (0.7–4.4)	0.194	1.4 (0.4–5.3)	0.631	0.3 (0.0–2.0)	0.192	0.6 (0.1–2.7)	0.468	NC		0.9 (0.1–8.0.1.0)	0.910	1.3 (0.3–6.5)	0.746
		Age (year)	1.1 (0.8–1.1)	0.568	1.2 (0.9–1.5)	0.200	1.0 (0.7–1.4)	0.909	1.0 (0.7–1.4)	0.798	NC		1.0 (0.7–1.6)	0.931	1.1 (0.8–1.6)	0.517
		Female sex	1.9 (0.8–4.5)	0.126	1.1 (0.3–3.5)	0.932	1.5 (0.4–5.3)	0.501	0.5 (0.1–1.9)	0.293	NC		3.6 (0.4–32.9)	0.255	0.6 (0.1–2.3)	0.416
		Prior COVID-19	1.2 (0.6–2.6)	0.612	1.2 (0.4–3.7)	0.717	1.4 (0.5–4.1)	0.559	**3.0 (1.3–6.8)**	**0.007**	NC		2.6 (0.8–8.3)	0.101	1.2 (0.3–4.3)	0.802
Nausea		Dose 2/Dose 1	NC		2.3 (0.6–9.6)	0.255	NC		NC		1.4 (0.2–13.7)	0.749	NC		4.5 (0.6–33.0)	0.138
		Age (year)	NC		**1.5** **(1.2–1.9)**	**0.002**	NC		NC		1.7 (0.9–3.4)	0.112	NC		1.2 (0.8–1.8)	0.275
		Female sex	NC		3.0 (0.6–14.9)	0.172	NC		NC		2.6 (0.3–25.0)	0.396	NC		2.8 (0.3–27.5)	0.367
		Prior COVID-19	NC		0.2 (0.0–3.9.0.9)	0.279	NC		NC		1.0 (0.2–6.7)	0.965	NC		1.0 (0.2–6.6)	0.138
Malaise		Dose 2/Dose 1	**2.5** **(1.3–4.9)**	**0.007**	4.8 (2.0–12.0)	0.001	2.3 (0.8–7.0.8.0)	0.132	0.8 (0.2–4.1)	0.804	1.6 (0.5–5.7)	0.435	0.3 (0.1–2.7)	0.309	2.8 (0.5–17.3)	0.261
		Age (year)	0.8 (0.7–1.0.7.0)	0.107	1.3 (1.1–1.5)	0.003	1.1 (0.8–1.5)	0.482	1.1 (0.8–1.5)	0.466	1.2 (0.8–1.7)	0.369	1.2 (0.9–1.5)	0.255	1.2 (0.9–1.7)	0.259
		Female sex	**3.3** **(1.5–7.0.5.0)**	**0.002**	1.4 (0.5–3.9)	0.494	3.0 (0.8–11.0)	0.094	0.8 (0.2–3.4)	0.817	2.7 (0.6–11.6)	0.174	4.4 (1.0–20.2.0.2)	0.057	1.3 (0.2–8.2)	0.751
		Prior COVID-19	1.0 (0.5–2.1)	0.911	1.6 (0.8–3.5)	0.197	1.1 (0.4–3.1)	0.861	1.7 (0.6–4.9)	0.364	1.4 (0.5–4.1)	0.520	0.7 (0.2–2.9)	0.664	1.6 (0.4–6.1)	0.488
Chills		Dose 2/Dose 1	NC		1.2 (0.2–5.8)	0.839	NC		NC		2.9 (0.2–47.6)	0.459	NC		NC	
		Age (year)	NC		1.2 (1.0–1.6.0.6)	0.079	NC		NC		1.0 (0.5–2.2)	0.980	NC		NC	
		Female sex	NC		6.9 (0.9–55.2)	0.068	NC		NC		1.1 (0.1–19.6)	0.927	NC		NC	
		Prior COVID-19	NC		0.9 (0.2–3.7)	0.851	NC		NC		4.5 (0.9–22.2)	0.064	NC		NC	
Headache		Dose 2/Dose 1	1.3 (0.7–2.3)	0.408	1.3 (0.8–3.0.8.0)	0.513	1.0 (0.4–2.5)	0.932	0.8 (0.3–2.5)	0.721	2.2 (0.8–6.3)	0.137	1.3 (0.5–3.5)	0.669	1.4 (0.4–4.2)	0.603
		Age (year)	1.0 (0.8–1.1)	0.845	1.0 (0.8–1.2)	0.931	1.0 (0.8–1.2)	0.793	1.0 (0.8–1.3)	0.927	1.1 (0.8–1.4)	0.688	1.0 (0.8–1.3)	0.890	1.2 (0.9–1.5)	0.213
		Female sex	**2.0** **(1.1–3.5)**	**0.017**	**4.7 (1.8–12.4)**	**0.001**	1.9 (0.8–4.4)	0.150	2.0 (0.7–5.8)	0.195	1.2 (0.4–3.3)	0.734	2.1 (0.8–5.5)	0.131	0.8 (0.3–2.0.3.0)	0.561
		Prior COVID-19	1.0 (0.6.1.8)	0.970	1.1 (0.6–2.3)	0.755	1.1 (0.5–2.4)	0.876	2.3 (1.1–4.5)	0.019	1.0 (0.4–2.7)	0.967	0.6 (0.2–2.1)	0.423	0.3 (0.1–2.2)	0.256
Joint Pain	Dose 2/Dose 1		0.9 (0.3–3.2)	0.866	1.3 (0.4–5.1)	0.655	1.6 (0.4–6.6)	0.555	0.3 (0.0–2.5.0.5)	0.257	2.3 (0.5–10.5)	0.278	0.8 (0.2–4.0.2.0)	0.830	NC	
	Age (year)		1.0 (0.8–1.4)	0.873	**1.2** **(1.0–1.5.0.5)**	**0.047**	0.9 (0.6–1.4)	0.686	0.9 (0.6–1.3)	0.528	1.0 (0.6–1.5)	0.899	1.2 (1.0–1.5.0.5)	0.062	NC	
	Female sex		1.4 (0.5–3.6)	0.524	4.2 (0.9–19.1)	0.066	6.5 (0.8–51.9)	0.076	0.8 (0.2–3.5)	0.724	1.4 (0.3–6.5)	0.641	1.4 (0.4–5.0.4.0)	0.574	NC	
	Prior COVID-19		1.2 (0.5–3.0.5.0)	0.685	1.7 (0.7–4.2)	0.255	0.7 (0.1–4.4)	0.696	**5.2 (2.2–12.0)**	**< 0.001**	1.9 (0.7–5.6)	0.225	1.5 (0.6–3.9)	0.830	NC	
Myalgia	Dose 2/Dose 1		2.2 (0.9–5.0.9.0)	0.074	2.2 (0.8–6.0.8.0)	0.132	NC		0.5 (0.1–2.4)	0.377	1.1 (0.3–4.1)	0.894	1.1 (0.2–5.1)	0.948	NC	
	Age (year)		1.2 (1.0–1.5.0.5)	0.119	**1.2** **(1.0–1.5.0.5)**	**0.021**	NC		1.1 (0.9–1.4)	0.297	1.0 (0.7–1.3)	0.776	**1.3 (1.1–1.6)**	**0.009**	NC	
	Female sex		1.4 (0.6–3.2)	0.380	2.8 (0.9–8.7)	0.067	NC		1.0 (0.3–3.2)	0.964	0.6 (0.2–2.1)	0.422	7.5 (0.9–60.0)	0.057	NC	
	Prior COVID-19		0.6 (0.2–1.8)	0.375	1.2 (0.5–2.9)	0.689	NC		**3.3 (1.6–6.9)**	**0.002**	2.1 (0.9–4.6)	0.083	0.7 (0.2–3.1)	0.616	NC	
Fatigue	Dose 2/Dose 1		1.5 (0.8–2.8)	0.224	1.7 (0.7–3.8)	0.215	NC		0.5 (0.1–2.3)	0.373	1.7 (0.5–5.9)	0.438	0.3 (0.0–2.6.0.6)	0.289	1.6 (0.5–5.0.5.0)	0.459
	Age (year)		0.9 (0.7–1.1)	0.175	1.0 (0.8–1.2)	0.699	NC		1.0 (0.7–1.4)	0.994	1.4 (0.9–2.1)	0.167	1.1 (0.9–1.4)	0.299	1.0 (0.7–1.3)	0.795
	Female sex		2.0 (1.0–4.1.0.1)	0.056	1.6 (0.8–3.5)	0.222	NC		0.7 (0.2–2.5)	0.627	1.0 (0.3–4.3)	0.966	1.7 (0.5–5.6)	0.407	1.3 (0.5–3.8)	0.616
	Prior COVID-19		1.1 (0.5–2.2)	0.796	1.7 (1.0–3.1.0.1)	0.063	NC		2.1 (0.9–4.9)	0.088	1.3 (0.3–4.6)	0.732	1.8 (0.8–4.2)	0.185	1.1 (0.4–3.0.4.0)	0.907

*NC* Not converged

**Table 4:** Incidence of local reactogenicities within the first day following sinopharm vaccination, by age, sex, and dose group

Variables		First dose			Second dose		
		*N*	*n* (%)	*P* value†	*N*	*n* (%)	*P* value†
Sex	Male	470	126 (26.8)	< 0.001	97	47 (48.5)	0.064
	Female	475	186 (39.2)		87	54 (62.1)	
Age group	10–11	47	22 (46.8)	0.110	13	6 (46.2)	0.647
	12–13	369	128 (34.7)		80	48 (60.0)	
	14–15	300	95 (31.7)		53	27 (50.9)	
	16–17	229	67 (29.3)		38	20 (52.6)	
Total		945	211 (27.7)	-	184	101 (54.9)	-

† Chi squared test

**Table 5:** Incidence of systemic reactogenicities within the first day following sinopharm vaccination, by age, sex, and dose group

Variables		First dose			Second dose		
		*N*	*n* (%)	*P* value†	*N*	*n* (%)	*P* value†
Sex	Male	470	42 (8.9)	< 0.001	97	15 (15.5)	0.139
	Female	475	88 (18.5)		87	21 (24.1)	
Age group	10–11	47	10 (21.3)	0.277	13	4 (30.8)	0.103
	12–13	369	45 (12.2)		80	10 (12.5)	
	14–15	300	46 (15.3)		53	15 (28.3)	
	16–17	229	29 (12.7)		38	7 (18.4)	
Total		945	130 (13.8)	-	184	36 (19.6)	-

† Chi squared test

### The incidence rate of COVID-19 infection, hospitalization, and death

A total of 487 COVID-19 cases have been reported among 18,528 participants. The total follow-up time amounted to 1,686,664 person-days, resulting in an incidence rate of 28.9 (95% CI: 26.4–31.6) per 100,000 person-days (Table 6). Among the COVID-19 patients, seven required hospitalization, but there were no reported cases of ICU admissions or deaths related to COVID-19.

**Table 6:** The incidence rates (per 100,000 participants) of COVID-19 infection by sex and age groups

Variables			No (%)	Incidence rate (95% CI)
Total			487 (100)	28.9 (26.4–31.6)
Sex		Male	234 (48.1)	28.1 (24.7–31.9)
		Female	253 (51.9)	29.6 (26.2–33.5)
Age groups (Year)	5–8		49 (10.1)	13.4 (10.1–17.7)
	9–11		52 (10.7)	15.7 (11.9–20.6)
	12–14		236 (48.5)	38.8 (34.1–44.0)
	15–18		150 (30.8)	39.5 (33.7–46.4)

The multivariable Cox proportional hazard regression for the occurrence of COVID-19 infection after vaccination showed that allergies and chronic respiratory diseases increased the hazard of COVID-19 infection, while the administration of a second dose of the vaccine reduced the hazard by half (Hazard Ratio: 0.51). A history of COVID-19 infection had no effect on re-infection after vaccination (Table 7).

**Table 7:** The association of demographics, and underlying diseases on COVID-19 infection in multiple Cox regression, stratified on study sites

Independent Variables	*n*	Number of events	Person time (day)	Unadjusted (simple model)		Adjusted (multiple model)	
				Hazard ratio (95% CI)	*P* Value	Hazard ratio (95% CI)	*P* Value
Age (Year)	18,528	487	1,686,664	1.12 (1.09–1.16)	< 0.001	1.13 (1.10–1.17)	< 0.001
Female gender	9370	253	853,437	1.05 (0.88–1.25)	0.602	1.08 (0.90–1.29)	0.424
Prior COVID-19 disease	1854	72	174,335	1.34 (1.04–1.73)	0.024	1.26 (0.97–1.62)	0.080
Respiratory	122	7	10,766	2.37 (1.12–5.02)	0.024	2.25 (1.06–4.78)	0.034
Cardiac	51	3	4587	2.46 (0.79–7.66)	0.121	2.10 (0.67–6.57)	0.200
Allergy	258	11	23,242	1.63 (0.90–2.97)	0.109	1.68 (092–3.07)	0.091
Received second dose	10,642	333	1,108,926	0.51 (0.39–0.66)	< 0.001	0.45 (0.35–0.59)	< 0.001

### Serious adverse events and adverse events of special interests

During the follow-up period, 91 participants were admitted to hospitals. Among the hospitalized individuals, two cases were recognized as AESI, including a generalized convulsion in a 13-year-old girl, 33 days after the first dose of the vaccine, and diabetic ketoacidosis (DKA) in a 17-year-old boy, 13 days after the second dose of the vaccine. According to the decisions of the classification committee, the hospitalization of other participants was not related to COVID-19 vaccination, and none of the participants were diagnosed as SAE cases.

## Discussion

This CEM study with the participation of 18,528 children and adolescents aged aged 5 to 18 Years showed that the SARS-CoV-2 Vaccine (Vero Cell), Inactivated (lnCoV), was a safe and effective vaccine for COVID-19 disease. We did not find any SAE. The two cases of DKA and generalized convulsion (1.08 cases per 10,000 vaccinated individuals) were anticipated, considering the annual incidence rates of convulsion (6.2 per 10,000) [13, 14] and DKA [17] documented in prior studies involving children and adolescents.

The reported case of DKA occurred in a patient with a known history of diabetes. Potential triggers for DKA following vaccination could include an intense inflammatory response or a coincidental, unrelated intercurrent illness. The differential diagnosis is straightforward, based on the presence of hyperglycemia and ketoacidosis in a diabetic patient. This case highlights the importance of considering diabetes—including new-onset diabetes—in patients presenting with gastrointestinal symptoms following vaccination. It further suggests that vaccination teams should be aware of this potential, though rare, association. Several case reports have described the onset of autoimmune diabetes and DKA following COVID-19 vaccination [19–23]. It is important to note, however, that this association is rare and remains unproven, with large population [24] and systematic review [25] studies showing no significant link. Furthermore, the risk of developing severe metabolic complications from SARS-CoV-2 infection itself is substantially greater. Therefore, while this temporal association is noted, it should not detract from the overwhelming public health benefit of COVID-19 vaccination.

The reported generalized convulsion was evaluated as a first-ever seizure. Key differential diagnoses include an epileptic seizure provoked by post-vaccination fever (resembling a febrile seizure, though these typically occur in children), syncope with accompanying myoclonic activity, or a manifestation of an underlying neurological disorder. The patient in this study underwent a neurological workup, which revealed no acute abnormalities; the event did not recur. This underscores the necessity of a complete medical evaluation for any seizure episode following vaccination to rule out other causes. Proactive monitoring for fever and advising on the use of antipyretics may help mitigate the risk of febrile seizures in susceptible individuals. Seizures and convulsions have been reported following COVID-19 infection in other studies [26, 27]. However, COVID-19 vaccination has not been associated with an increased risk of seizures [26].

In another study with one month of follow-up of 234 adolescents who were vaccinated with Sinopharm, no SAE were reported [10]. However, Thonginnetra et al. reported three cases of neurological events, four cases of palpitation, one case of myocarditis, one case of anaphylaxis, and one case of herpes zoster in a 30-day follow-up of 22,224 adolescents aged 10 to 17 years in Thailand [13]. The differences seen in these studies may possibly be due to differences in study design, follow-up period, sample size, and even racial differences. A low rate of SAE and myocarditis was also reported after receiving mRNA vaccines in 5 to 11 years old children [28].

The incidence and severity of local and systemic reactogenicities were also low, and with the exception of the pain at the injection site, other reactions occurred in less than 10% of participants. Other evidence also showed that inactivated vaccines had the lowest adverse events, while mRNA vaccines had the highest reactogenicity [29]. In contrast to our previous work on adults [30] and other studies [8, 10, 13, 31], which reported a higher incidence of reactogenicities after the first dose of vaccines, the incidence of the most local and systemic reactogenicities was not statistically different after the first and second doses of the vaccine. This finding is similar to the results of a systematic review and met-analysis [29]. However, other studies on Sinopharm [32, 33], CoronaVac [34], and mRNA vaccines [29, 34–39] in individuals over 18 years old reported a higher frequency of reactogenicities in the second dose compared to the first dose of vaccines. The pain at the injection site was the only adverse event that was more frequent after the second dose of Sinopharm in the first three days after vaccination. In the Tawinprai et al. study [10], which was conducted in the age group of 12 to 17 years, 18.1% of the participants reported pain at the injection site after the first dose and 12.4% of them after the second dose of Sinopharm. However, in the Thonginnetra et al. study [13], these numbers were 51.4% and 6.7%, respectively. The main reason for the difference in these estimates may be attributed to the differences in study design, the age group of participants, and the data collection method. For example, in the Thonginnetra et al. study [13], where there is a significant difference between the first and second doses in the incidence of pain at the injection site, the question about reactogenicities after the first dose was asked retrospectively at the time of receiving the second dose of the vaccine. This scenario also occurred in the Mafinezhad et al. study [8]. Despite the above two studies, in the present study, the daily data collection was done actively and by following an approved and recommended guideline [16]. In a clinical trial study investigating the safety of Sinopharm in people under 18 years of age [12], the local and systemic adverse effects were less frequent and, to a large extent, similar to our results. Similar to our results, a study in the UAE on people over 18 years of age receiving Sinopharm [40] and another prospective study [41] showed no difference in the occurrence of local and systemic adverse effects after the first and second doses of the vaccine. It should be noted that participants in the reactogenicity subset were between 10 and 18 years of age. Therefore, the results of current study are generalizable only to this age group.

The incidence of COVID-19 infection in vaccinated individuals was low at approximately 43% of the rate observed in Iranian adults [42]. The differences in study periods, the shapes and severity of the epidemic, and the age groups of participants are the primary reasons for the discrepancy between these two studies. The hazard ratio of COVID-19 infection was 0.45 in those who received two doses of the vaccine. This indicates that the incidence rate of COVID-19 among participants who received two doses of the vaccine is approximately half that of those who received only one dose. In line with our results, it was previously shown that partial vaccination with a single dose of Sinopharm did not protect against hospitalization and death [43]. In the current study, among the 10,642 participants who received the second vaccine dose, 333 (3.1%) contracted a COVID-19 infection within three months of vaccination. This incidence of breakthrough infections supports the use of booster doses to lower the infection rate and improve coverage against new viral variants. We expected a lower rate of COVID-19 infection in those with a prior history of COVID-19, but prior COVID-19 was not associated with reinfection. This issue may be attributed to different variants of SARS-CoV-2 during the study period, as most prior infections were due to the Delta variant and most reinfections occurred when the dominant variant of SARS-CoV-2 was Omicron. Students with a history of respiratory disease had a higher risk of COVID-19 infection. This may be partly due to the increased sensitivity of these individuals and their parents to disease, along with a higher frequency of COVID-19 testing (detection bias). Increased healthcare utilization and repeated COVID-19 testing—even for mild symptoms—may improve detection of subclinical cases but could also introduce bias, leading to false associations between general respiratory illnesses and COVID-19. Notably, studies report conflicting effects of pre-existing respiratory conditions on COVID-19 risk. For instance, allergic conditions in adults (≥ 18 years) have been linked to higher COVID-19 diagnosis rates [44], while a systematic review and meta-analysis found that inhaled corticosteroid use was inversely associated with COVID-19 infection [45]. These disparities may reflect differences in immune modulation or surveillance biases.

The main strengths of this study include using a robust approved guideline [16], the active surveillance that was conducted, the weekly follow-up of participants, and the investigation and classification of all hospitalized participants. However, the total sample size of the study did not reach 30,000 people based on the WHO recommendation, and only 57.4% of the participants received the second dose of the vaccine, which can be considered a limitation of our study for the true estimation of AESIs. The sample size for reactogenicity estimates did not reach 1,000 participants, especially for the second dose of the vaccine. This limitation results in wide confidence intervals for point estimates, particularly for rare reactogenicities. Additionally, the statistical power for comparing reactogenicity between vaccine doses may be limited (increasing the risk of type II error) due to small sample sizes in subgroup analyses. The age range for the reactogenicity subset was between 10 and 18 years, so the results cannot be generalized to younger children. It should also be noted that some children with COVID-19 may be asymptomatic; therefore, the reported rates of COVID-19 infection may be underestimated.

## Conclusion

COVID-19 vaccination with two doses of Sinopharm in children and adolescents aged 5 to 18 Years is safe and can be used for this population, especially for those with allergies and chronic respiratory diseases. The low reactogenicity rate (predominantly mild cases) along with low rates of SAEs and AESIs could support COVID-19 vaccination promotion in pediatric populations and help address vaccine hesitancy in future vaccination programs. While this study provides evidence of Sinopharm’s safety, further studies are needed, particularly in younger children and with longer follow-up durations. The occurrence of breakthrough infections following the two-dose primary series indicates that protection is not absolute. This supports the rationale for booster doses to sustain and enhance immunity against COVID-19. Consequently, further studies are warranted to comprehensively evaluate the safety of such booster strategies.

## Data Availability

All data of this study can be provided at the request of the corresponding author (Prof. Mohammad Hassan Emamian, via emamian@shmu.ac.ir). All researchers around the world can send their proposed titles. After screening by a scientific committee, the new titles will be approved and the required data will be available for researchers. The new articles and reports then will be prepared in collaboration with the researchers of this study.

## Abbreviations

CEM: Cohort Event Monitoring
WHO: World Health Organization
SAEs: Serious Adverse Events
AESIs: Adverse Events of Special Interest
SD: Standard Deviation
PCR: Polymerase Chain Reaction
